# Single-cell RNA-seq data analysis on the receptor ACE2 expression reveals the potential risk of different human organs vulnerable to 2019-nCoV infection

**DOI:** 10.1007/s11684-020-0754-0

**Published:** 2020-03-12

**Authors:** Xin Zou, Ke Chen, Jiawei Zou, Peiyi Han, Jie Hao, Zeguang Han

**Affiliations:** 1 Key Laboratory of Systems Biomedicine (Ministry of Education), Shanghai Centre for Systems Biomedicine, Shanghai Jiao Tong University, Shanghai, 200240 China; 2 Ruijin Hospital Affiliated to Shanghai Jiao Tong University School of Medicine, Shanghai, 200025 China

**Keywords:** 2019-nCoV, ACE2, single-cell RNA-seq

## Abstract

It has been known that, the novel coronavirus, 2019-nCoV, which is considered similar to SARS-CoV, invades human cells via the receptor angiotensin converting enzyme II (ACE2). Moreover, lung cells that have ACE2 expression may be the main target cells during 2019-nCoV infection. However, some patients also exhibit non-respiratory symptoms, such as kidney failure, implying that 2019-nCoV could also invade other organs. To construct a risk map of different human organs, we analyzed the single-cell RNA sequencing (scRNA-seq) datasets derived from major human physiological systems, including the respiratory, cardiovascular, digestive, and urinary systems. Through scRNA-seq data analyses, we identified the organs at risk, such as lung, heart, esophagus, kidney, bladder, and ileum, and located specific cell types (i.e., type II alveolar cells (AT2), myocardial cells, proximal tubule cells of the kidney, ileum and esophagus epithelial cells, and bladder urothelial cells), which are vulnerable to 2019-nCoV infection. Based on the findings, we constructed a risk map indicating the vulnerability of different organs to 2019-nCoV infection. This study may provide potential clues for further investigation of the pathogenesis and route of 2019-nCoV infection.

**Electronic Supplementary Material:**

Supplementary material is available for this article at 10.1007/s11684-020-0754-0 and is accessible for authorized users.

## Introduction

On January 30, 2020, the World Health Organization (WHO) declared the novel coronavirus 2019-nCoV outbreaks as a Public Health Emergency of International Concern [1]. Experts and scientists worldwide are working rapidly to expand scientific knowledge on this new virus. In addition to respiratory symptoms, such as cough and shortness of breath, clinical manifestations including multiple organ failure were also observed, which is unusual with other coronavirus infections.

The initial step of 2019-nCoV infection is its entrance into human cells. The analysis of Xu *et al.* [2] showed that the 2019-nCoV and the SARS-CoV share a common ancestor that resembles the bat coronavirus HKU9-1. These coronaviruses have very similar spike protein 3-D structures that are considered to have strong binding affinity to the human cell receptor, angiotensin-converting enzyme 2 (ACE2). Therefore, the cells with ACE2 expression may act as target cells and thus are susceptible to 2019-nCoV infection; such cells include type II alveolar cells (AT2) of the lungs [3]. Hence, we believe that the ACE2 expression pattern in different organs, tissues, and cell types could uncover the potential risk to 2019-nCoV infection because the target cells expressing ACE2 might permit coronavirus entry, multiplication, spread, and pathogenesis. Previously, the RNA and protein expressions of ACE2 were investigated using bulk samples from the heart, lung, kidney, and other organs. However, these bulk data only consider average expression and utterly ignore

*The authors wish it to be known that, in their opinion, the first three authors should be regarded as Joint First Authors.

cell diversity and specificity.

In addition to AT2 cells, the cell types and organs that express ACE2 as a potential receptor for 2019-nCoV are still unclear. Therefore, this study explores the existence of host cell receptor ACE2 in various cell types using published single-cell RNA-seq (scRNA-seq) data to track the potential 2019-nCoV infection in human organs. Based on these data, we construct a map of ACE2 expression over different cell types of different organs to predict the potential risk for 2019-nCoV infection and injury. This work can contribute to an improved understanding of how 2019-nCoV invades human body systems.

## Materials and methods

Here, we explored the published scRNA-seq datasets from various tissues and organs of different human body systems, including the respiratory system [4,5] (nasal mucosa, respiratory track, bronchus, and lung), the cardiovascular system [6] (heart), the digestive system [7–10] (esophagus, stomach, ileum, and liver), and the urinary system [11,12] (kidney and bladder). The lung scRNA-seq data were acquired from the Gene Expression Omnibus (GEO) database under the series number GSE122960; the nasal mucosa, respiratory track, bronchus scRNA-seq data were from GSE121600; the heart data were from GSE106118; the esophagus data were downloaded from https://www.tissuestabilitycellatlas.org/; the ileum data were from GSE134809 sample GSM3972018; the stomach data were from GSE134520 sample GSM3954949; the liver data were from GSE115469; the kidney data were from GSE109564; and the bladder data were from GSE129845 sample GSM3723358.

The ACE2 expression distribution across distinct cell types of different organs were evaluated, and then the cell types with high ACE2 expression levels were identified in accordance with the scRNA-seq datasets. A generally accepted consensus suggests that the 2019-nCoV virus tends to attack lung AT2 cells via host receptor ACE2, and the expression level of ACE2 in AT2 cells were thus used as a reference. We defined that any type of cells with a proportion of ACE2 positive cells (UMI count $> 0$) comparable to or larger than that of AT2 cells is highly vulnerable to 2019-nCoV infection, and the corresponding organs should be listed as highly risky.

We used Seurat V3.0 to discriminate different cell types. The data were first normalized using the LogNormalize method, and the cell clustering performance were conducted using the top 2000 most variable genes with the resolution set to 0.5 for kidney and 0.1 for the others. The cell scatter plots were obtained using the UMAP method [13].

## Results

### Pulmonary AT2 cells and respiratory epithelial cells exhibit high ACE2 expression

We analyzed scRNA-seq datasets from nasal mucosa, respiratory track, bronchus, and lung of the respiratory system. To construct the reference, we first calculated the proportion of ACE2-positive lung AT2 cells from eight individuals. For each individual, the clusters of lung AT2 cells were identified in accordance with the coexpression of ACE2, SFTPB, and SFTPC genes (Fig. S1). The average proportion of ACE2-positive AT2 cells among the eight individuals was approximately 1% with 1% standard derivation. We thus defined the cell types with a $>$ 1% proportion of ACE2 positive cells as high risk and those with a $<$ 1% proportion ACE2 positive cells as low risk.

Interestingly, the respiratory epithelial cells from the respiratory track sample contained approximately 2% ACE2 positive cells, and the respiratory track is thus regarded as high risk (Fig. 1). However, almost no cell obtained from the nasal and bronchial samples showed high ACE2 expression (Figs. S2 and S3), and such samples are therefore low risk.

### Myocardial cells with high ACE2 expression

The scRNA-seq data from human heart showed that more than 7.5% myocardial cells have positive ACE2 expression (Fig. 2), thereby implying that heart could be in high risk of 2019-nCoV infection, especially in the presence of the virus in blood or viremia.

### Ileum and esophagus epithelial cells have high ACE2 expression

We explored the scRNA-seq data from the digestive system, including esophagus, stomach, ileum, and liver. Interestingly, extremely high ACE2 expression was found in ileal epithelial cells (~30% ACE2-positive cells, Fig. 3); thus, ileum could be at high risk. More than 1% ACE2 positive esophagus epithelial cells were found, and esophagus can thus be regarded as high risk (Fig. 4). By contrast, the cells from stomach and liver showed lower ACE2 expression levels ( $<$ 1% ACE2 positive cells, Figs. S4 and S5).

### Proximal tubule cells of kidney and bladder urothelial cells express ACE2

We also analyzed the scRNA-seq data from the urinary system, specifically kidney and bladder. Significantly high ACE2 expression was found in kidney proximal tubule (PT) cells with the proportion of ACE2-positive cells at approximately 4% (Fig. 5). Furthermore, the proportion of

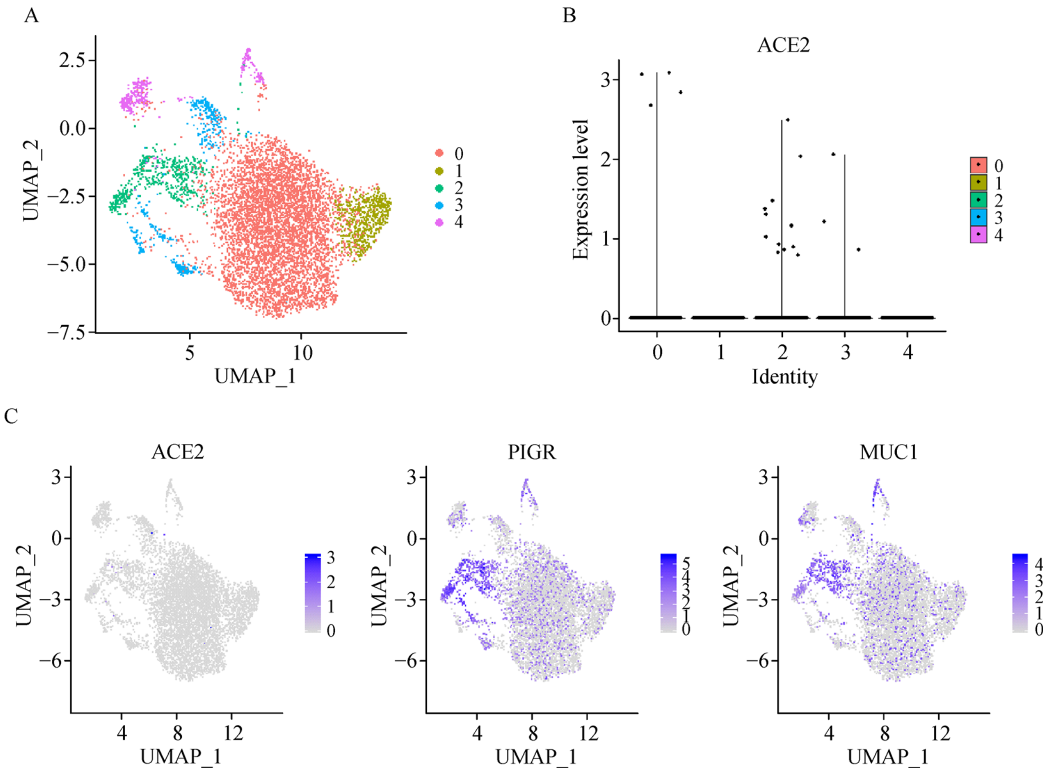

**Fig. 1** Respiratory track scRNA-seq data analysis showed epithelial cells with high ACE2 expression levels. (A) The cells were categorized into five clusters. (B) Violin plot of the ACE2 expression distribution of different cell clusters. (C) Scatter plots revealed that the cluster of cells with ACE2 expression also expressed canonical markers of respiratory epithelial cells PIGR and MUC1.

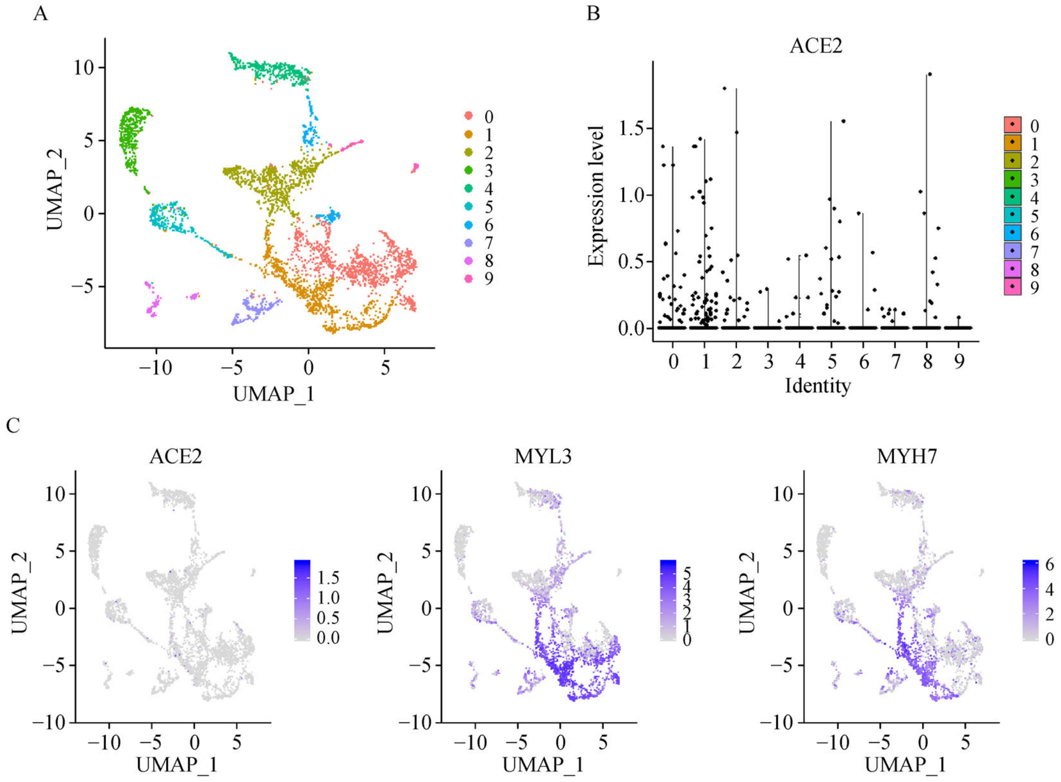

**Fig. 2** Heart scRNA-seq data analysis revealed myocardial cells with high ACE2 expression levels. (A) The cells were categorized into 10 clusters. (B) Violin plot of the ACE2 expression distribution of different cell clusters. (C) Scatter plots of the cluster of cells with high ACE2 expression levels also expressed canonical markers of myocardial cells MYL3 and MYH7.

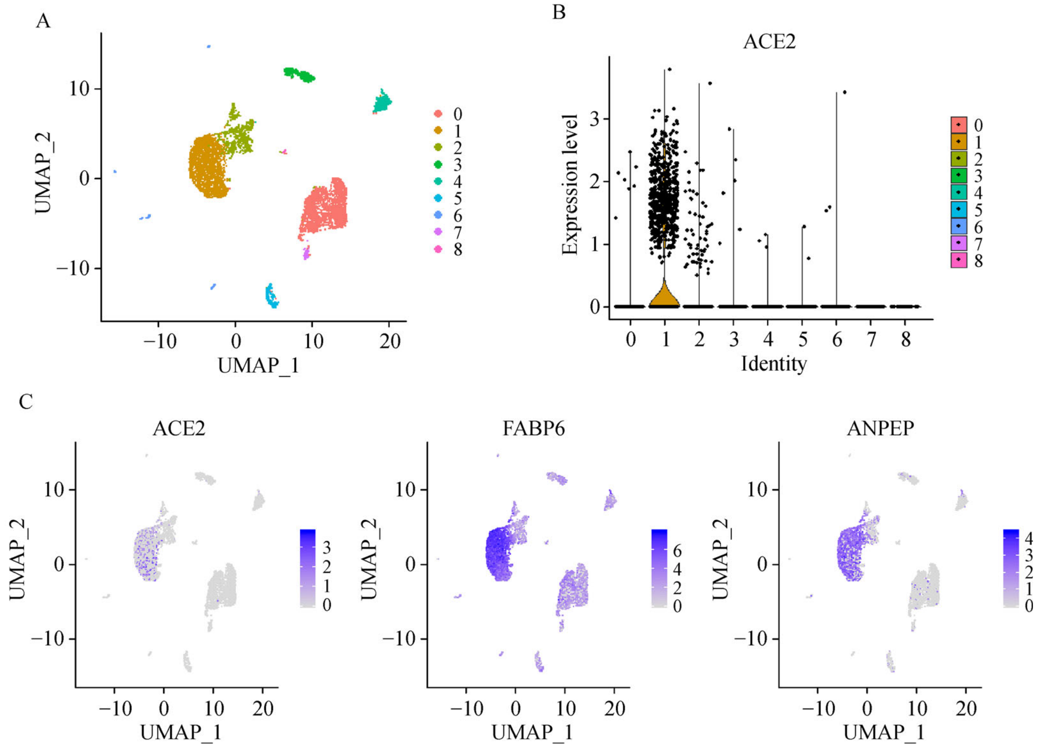

**Fig. 3** ScRNA-seq data analysis showed that ileal epithelial cells have high ACE2 expression. (A) The cells were categorized into nine clusters. (B) Violin plot of the ACE2 expression distribution of different cell clusters. (C) Scatter plots showed that the cluster of cells with high ACE2 expression also expressed canonical markers of ileal epithelial cells, FABP6, and ANPEP.

ACE2-positive cells in the bladder urothelial cells was 2.4% (Fig. 6). Therefore, kidney and bladder were considered high risk, especially in viremia.

On the basis of the above findings, a 2019-nCoV infection-related risk map of different organs can be constructed (Fig. 7). As a clinical implication, lung should be listed as having the highest risk. Interestingly, in addition to lung AT2, more than 1% of the cell population of some cell types from respiratory track, kidney, esophagus, bladder, ileum, and heart also expressed ACE2, and thus, those organs should be regarded as high risk for potential 2019-nCoV infection. This finding may explain the non-respiratory symptoms observed in 2019-nCoV pneumonia patients.

## Discussion

This study provides an overview of 2019-nCoV infection-related vulnerable organs using state-of-art single-cell techniques. According to scRNA-seq datasets, we managed to stratify the organs into high and low risk on the basis of their ACE2 expression levels in some cell types for the first time. The reported clinical symptoms of 2019-nCoV infection (e.g., dyspnea, diarrhea, acute cardiac injury, and kidney failure) might be related to the invasion of the novel coronavirus in the lung, upper respiratory track, ileum, heart, and kidney, especially in the presence of viremia.

In this study, we identified the organs with high and low vulnerability according to ACE2 expression. However, we admit that the 2019-nCoV invasion is not just about ACE2, and this observation means that our study may not have exhausted all 2019-nCoV vulnerable organs. In addition, given the gene expression variation between individuals, further study is still required to exclude the susceptibility of the organs categorized as low risk.

The mRNA expression of the receptor ACE2 indicates the transcription of the gene in these tissues and relevant cell types. To strengthen the expression pattern of ACE2, we also analyzed the protein expression of ACE2 in the stated human organs and cells by examining the Human Protein Atlas (https://www.proteinatlas.org), the UniProt (https://www.uniprot.org) databases, and some literatures. The resulting data showed that ACE2 protein is enriched in the enterocytes of small intestine and the renal tubules, as well as the lung alveolar epithelial cells, the heart cells, the arterial smooth muscle cells, and the gastrointestinal

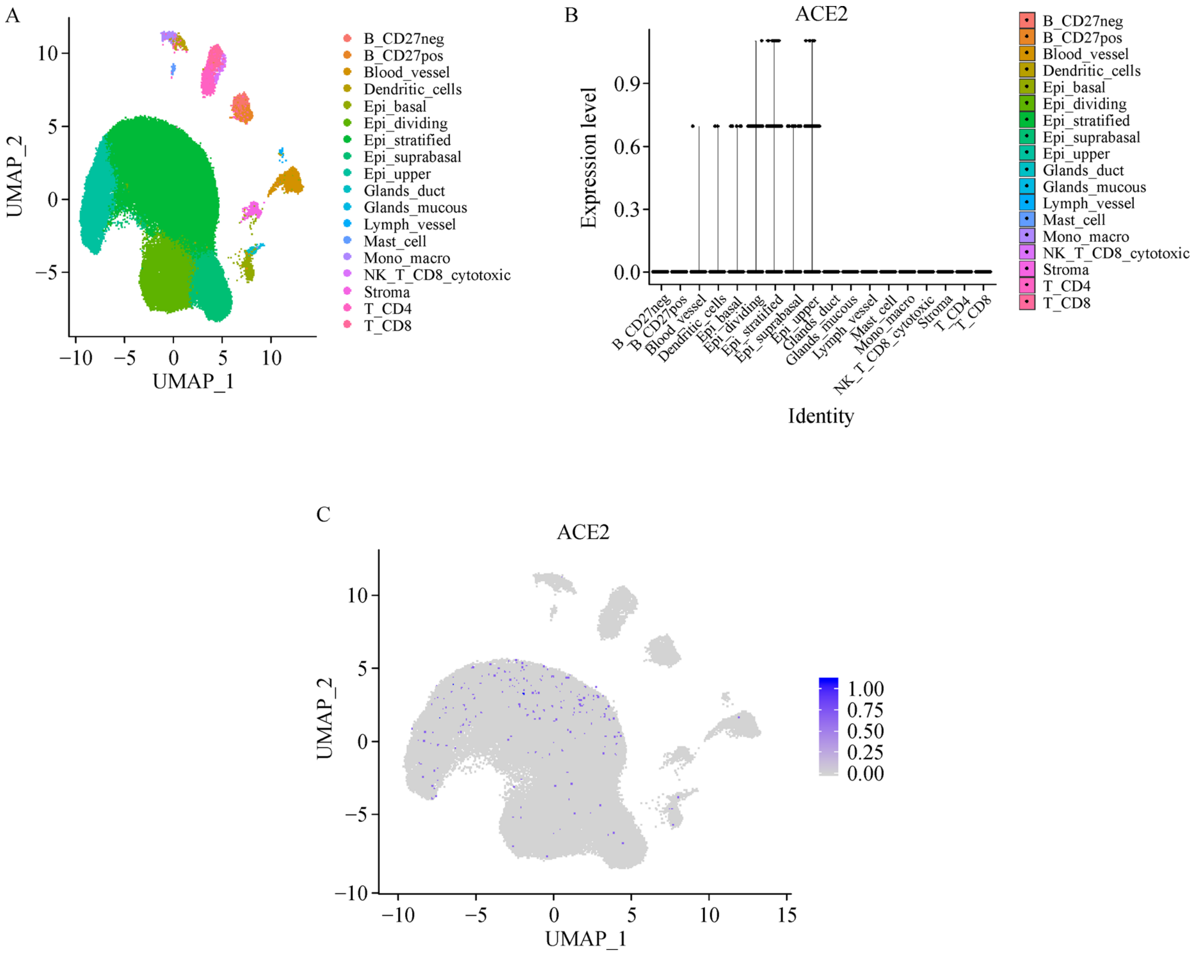

**Fig. 4** Esophagus scRNA-seq data analysis revealed that a few esophageal epithelial cells express ACE2. (A) The cells were categorized into 18 cell types. (B) Violin plot of the ACE2 expression distribution of different cell clusters. (C) ACE2 expression distribution demonstrated in a scatter plot.

system, as confirmed by published reports [14–18]. Moreover, myocardial infarction may increase ACE2 expression in heart, thereby suggesting that ACE2 plays an important role in cardiac injury [19]. The information on ACE2 protein expression is consistent with our results and thus supports the predictive risk map of different organs and tissues according to scRNA-seq data.

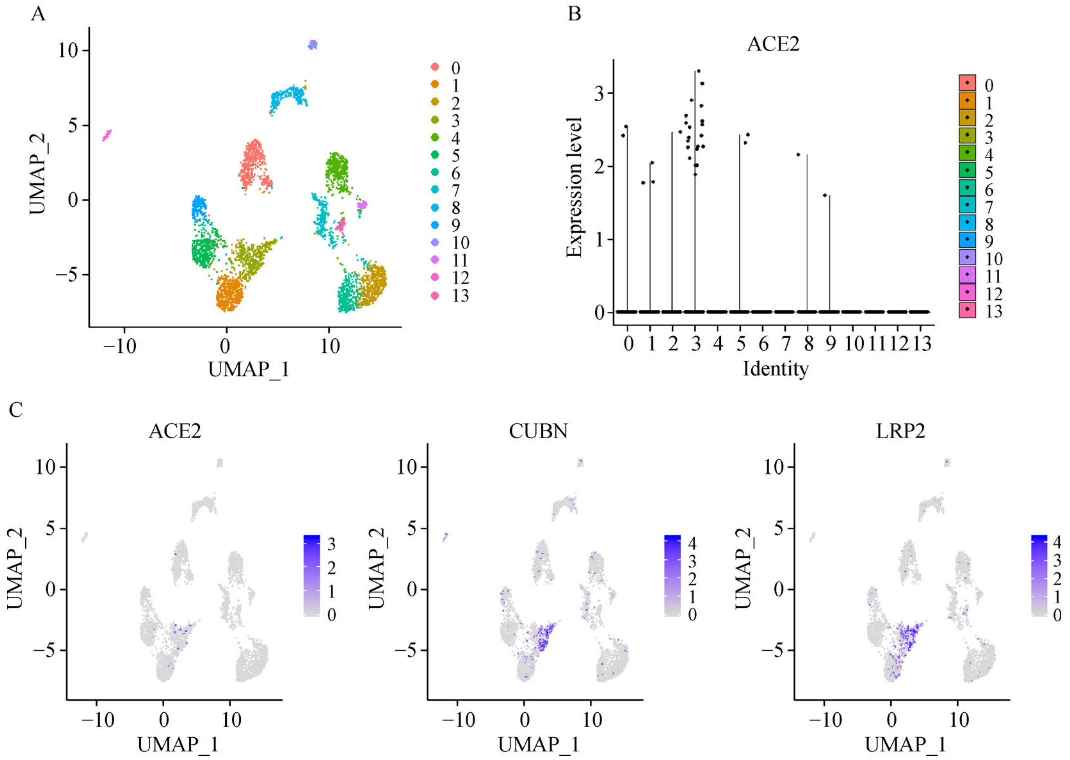

**Fig. 5** Kidney scRNA-seq data analysis revealed that the proximal tubule (PT) cells highly express ACE2. (A) The cells were categorized into 14 clusters. (B) Violin plot of the ACE2 expression distribution of different cell clusters. (C) Scatter plots showed that the cluster of cells with high ACE2 expression also expressed canonical markers of kidney PT cells CUBN and LRP2.

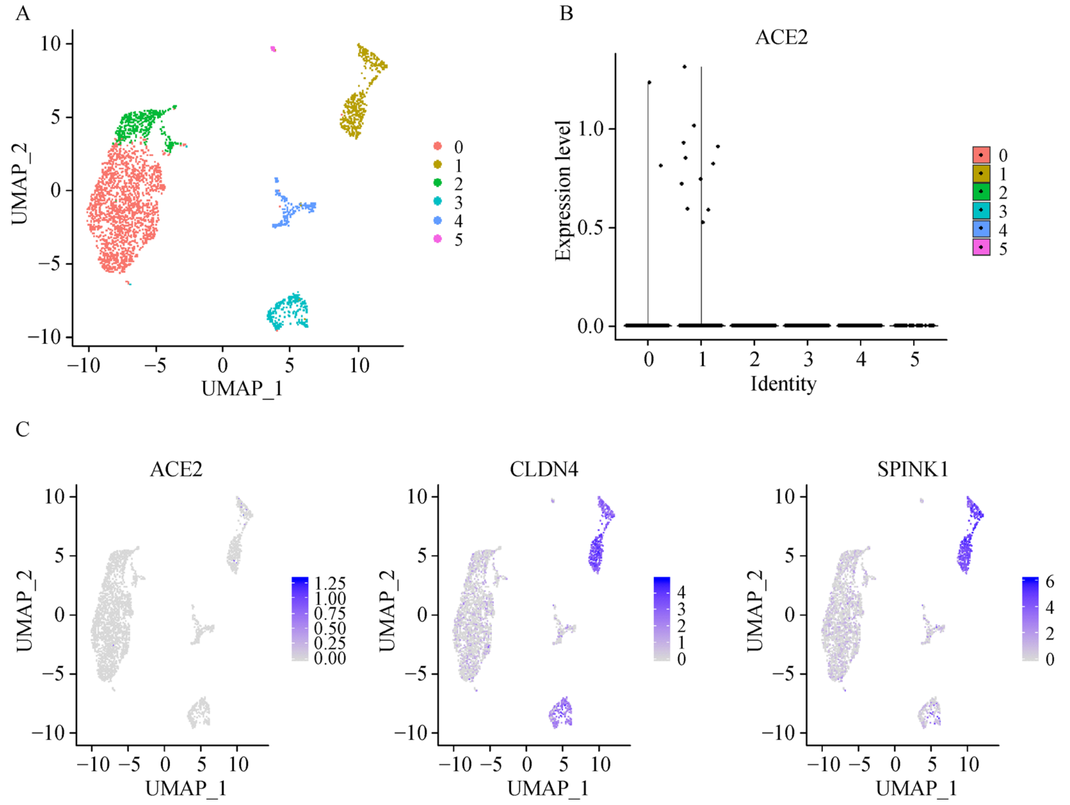

**Fig. 6** Bladder scRNA-seq data analysis showed that a few cells express ACE2. (A) The cells were categorized into six clusters. (B) Violin plot of the ACE2 expression distribution of different cell clusters. (C) Scatter plots showed that the cluster of cells with high ACE2 expression also expressed canonical markers of bladder urothelial cells CLDN4 and SPINK1.

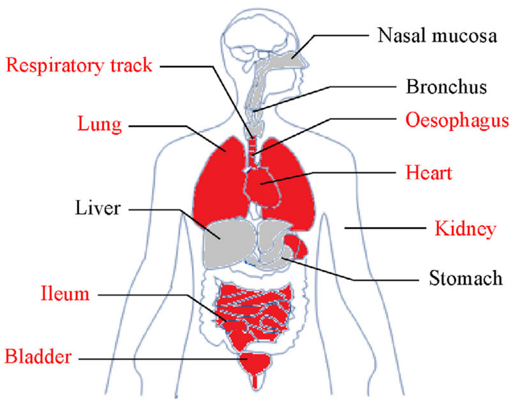

**Fig. 7** 2019-nCoV infection-related vulnerable organs with high risk are highlighted in red; low-risk organs are indicated in gray.

## Acknowledgements

This work was supported in part by the China National Science and Technology Major Project for Prevention and Treatment of Infectious Diseases (No. 2017ZX10203207 to Z.-G. H.), National Natural Science Foundation of China (No. 81672772 to Z.-G. H., No. 31601070 to J. H., No. 31800253 to K. C.), Interdisciplinary Program of Shanghai Jiao Tong University (Nos. 2019TPA09 and ZH2018ZDA33 to Z.-G. H., J. H., and X. Z.), Shanghai Sailing Program (No. 17YF1410400 to K. C.) and Innovative Research Team of High-Level Local Universities in Shanghai.

## Compliance with ethics guidelines

Xin Zou, Ke Chen, Jiawei Zou, Peiyi Han, Jie Hao, and Zeguang Han declare that they have no conflict of interest. This manuscript does not involve a research protocol requiring approval by the relevant institutional review board or ethics committee.

**Electronic Supplementary Material** Supplementary material is available in the online version of this article at https://doi.org/10.1007/s11684-020-0754-0 and is accessible for authorized users.

**Open Access** This article is licensed under a Creative Commons Attribution 4.0 International License, which permits use, sharing, adaptation, distribution and reproduction in any medium or format, as long as you give appropriate credit to the original author(s) and the source, provide a link to the Creative Commons license, and indicate if changes were made.

The images or other third party material in this article are included in the article's Creative Commons license, unless indicated otherwise in a credit line to the material. If material is not included in the article's Creative Commons license and your intended use is not permitted by statutory regulation or exceeds the permitted use, you will need to obtain permission directly from the copyright holder.

To view a copy of this license, visit https://creativecommons.org/licenses/by/4.0/.

## Electronic Supplementary Material

Supplementary material, approximately 403 KB.
