# Supplementary material for: Single-cell RNA-seq data analysis on the receptor ACE2 expression reveals the potential risk of different human organs vulnerable to 2019-nCoV infection
Source: Front Med. 2020 Mar 12;14(2):185–92. doi: 10.1007/s11684-020-0754-0 (PMC7088738; doi:10.1007/s11684-020-0754-0)
Supplement: Supplementary file 1 — Supplementary material, approximately 403 KB. [file 11684_2020_754_MOESM1_ESM.pdf]

Electronic Supplementary Material Supplementary material is available in the online version of this article at https://doi.org/10.1007/s11684-020-0754-0 and is accessible for authorized users.

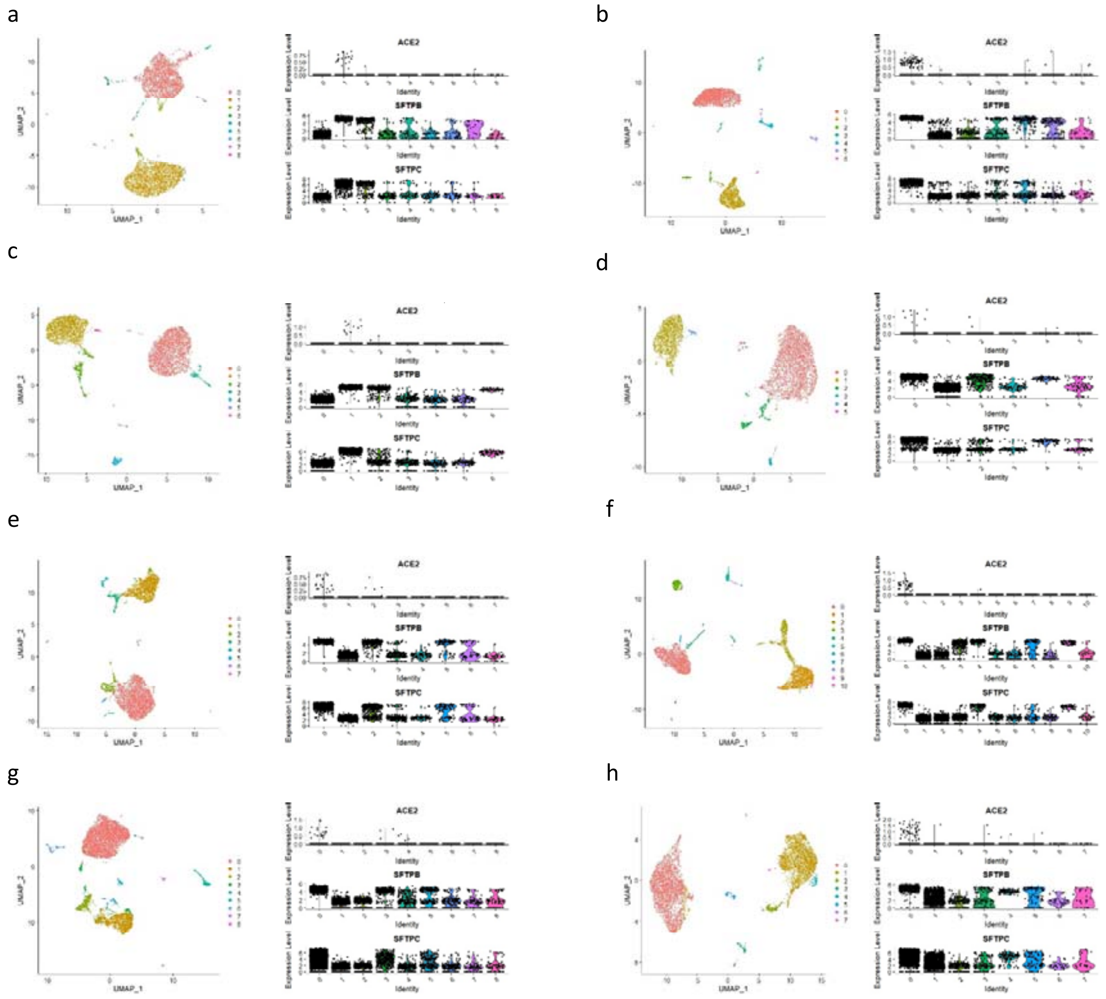

Fig. S1 The lung scRNA-seq data analysis results of 8 donors (a-h). For each donor, the cell cluster co-expressing ACE2, SFTPB and SFTPC are identified as AT2.

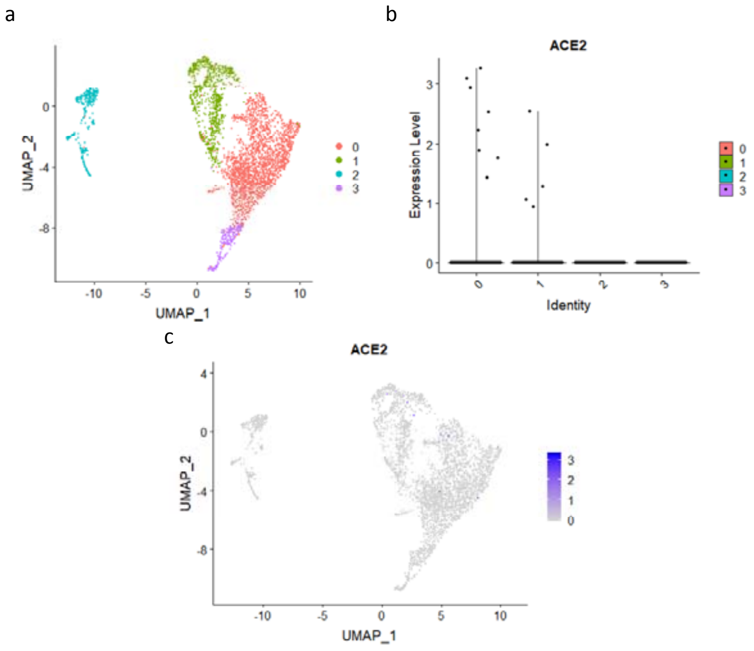

Fig. S2 Nasal mucosa scRNA-seq data analysis showed few cells expressing ACE2. a) The cells were categorized into 4 clusters. b) Violin plot showing the ACE2 expression distribution of different cell clusters. c) The ACE2 expression distribution demonstrated in scatter plot.

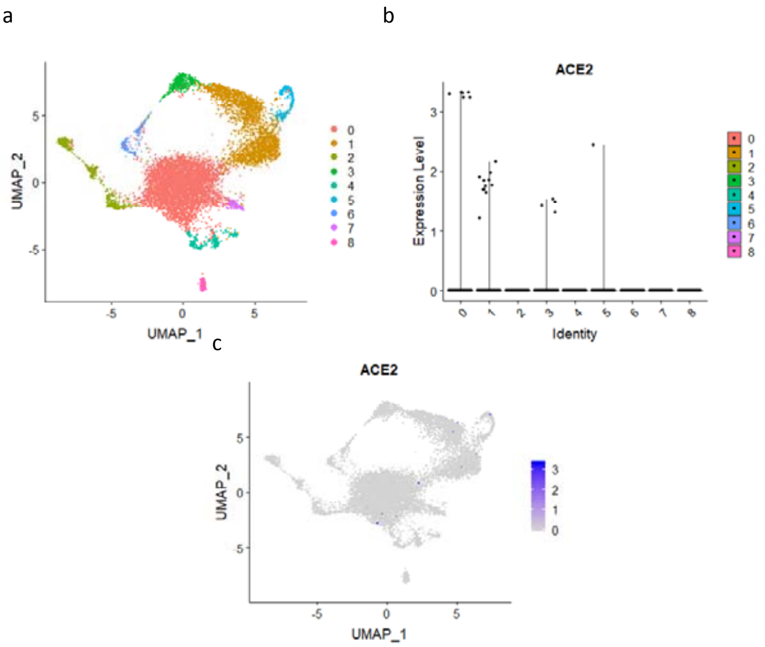

Fig. S3 Bronchus scRNA-seq data analysis showed few cells expressing ACE2. a) The cells were categorized into 4 clusters. b) Violin plot showing the ACE2 expression distribution of different cell clusters. c) The ACE2 expression distribution demonstrated in scatter plot.

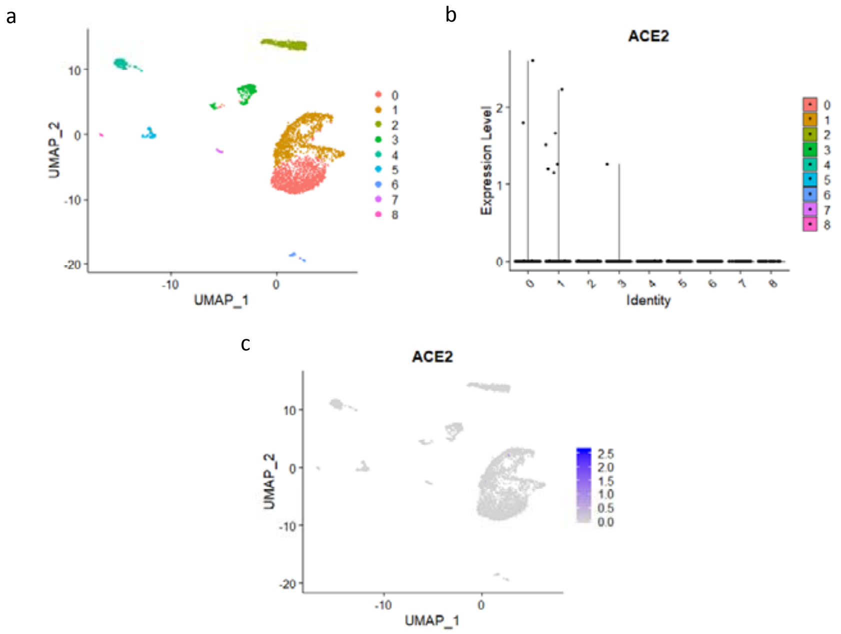

Fig. S4 Stomach scRNA-seq data analysis showed very few cells expressing ACE2. a) The cells were categorized into 9 cell types. b) Violin plot showing the ACE2 expression distribution of different cell clusters. c) The ACE2 expression distribution demonstrated in scatter plot.

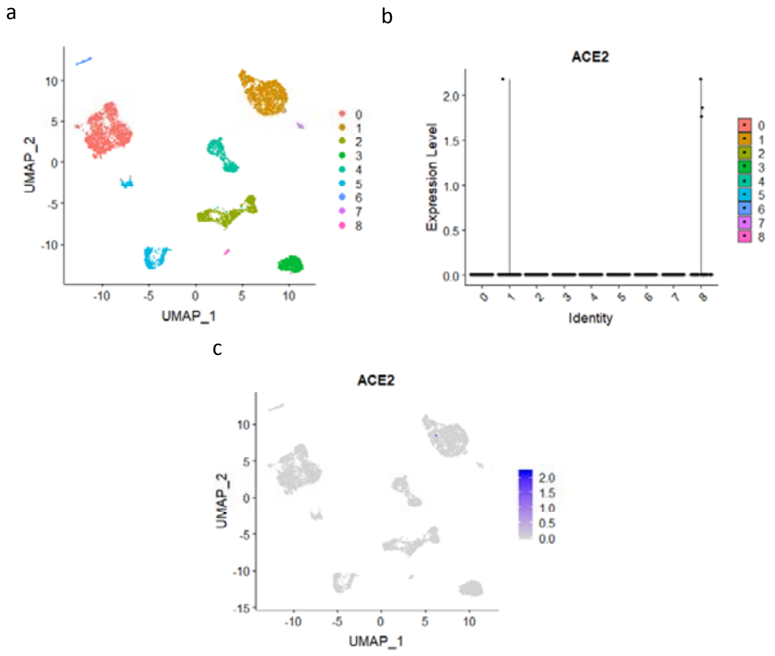

Fig. S5 Liver scRNA-seq data analysis showed almost no cell expressing ACE2. a) The cells were categorized into 9 cell types. b) Violin plot showing the ACE2 expression distribution of different cell clusters. c) The ACE2 expression distribution demonstrated in scatter plot.
